# Correction: Network Structure and Biased Variance Estimation in Respondent Driven Sampling

**DOI:** 10.1371/journal.pone.0148006

**Published:** 2016-01-22

**Authors:** Ashton M. Verdery, Ted Mouw, Shawn Bauldry, Peter J. Mucha

The following information is missing from the Funding section: Publication of this article was funded in part by The Pennsylvania State University Libraries Open Access Publishing Fund.
